# A Two-Dimensional Model for Pin-Load Distribution and Failure Analysis of Composite Bolted Joints

**DOI:** 10.3390/ma14133646

**Published:** 2021-06-30

**Authors:** Binkai Li, Yu Gong, Hao Xiao, Yukui Gao, Enquan Liang

**Affiliations:** 1 School of Aerospace Engineering and Applied Mechanics, Tongji University, Zhangwu Road 100#, Shanghai 200092, China; 1810398@tongji.edu.cn; 2 College of Aerospace Engineering, Chongqing University, Chongqing 400044, China; 3 Shanghai Aircraft Design and Research Institute, Jinke Road 5188#, Shanghai 201210, China; xiaohao@comac.cc (H.X.); liangenquan@comac.cc (E.L.); 4 School of Materials Science and Engineering, Tongji University, Caoan Road 4800#, Shanghai 201804, China; 12088@tongji.edu.cn; 5 Shanghai Key Laboratory of R&D for Metallic Function Materials, Tongji University, Caoan Road 4800#, Shanghai 201804, China

**Keywords:** composite laminated plate, pin load, joint strength, failure mode

## Abstract

Multibolt composite joints are widely used in aircraft structures. The determination of the pin-load distribution among the bolts is a critical step in the failure prediction of bolted joints. In this paper, a two-dimensional model of the multibolt composite structure is established for the pin-load distribution analysis. Its accuracy is validated by experimental results and the results from a 3D finite element model. Based on the determined pin-load distribution, FE models for a laminated plate with three-row fastener joints are built for failure prediction. Hashin stress criteria and the degradation guidelines of the material stiffness with respect to the different failure modes proposed by Tserpes are applied for the failure evaluation and the material degradation, respectively. The failure location and ultimate load are well predicted, which further validates the effectiveness and applicability of the proposed model for the pin-load distribution analysis.

## 1. Introduction

Due to the high specific strength and specific stiffness, composite materials are widely employed in the aerospace field [1]. Currently, composite materials are being used more and more in the main bearing structures, in preference to metals. As is well known, adhesive joint and mechanical joint are the two major connecting types for composite structures [2]. Adhesive joints are mainly used in nonessential bearing structures, transferring uniform loading. Mechanical joints are mainly used for components that transfer concentrated loads requiring high reliability. It is well known that the joint for important load bearing is dominated by mechanical joints with multirow pins. In general, there are four main methods for the determination of the load distribution in composite mechanical joints with multipin connection, namely, experimental [3], analytical [4,5], spring-based stiffness [6,7,8,9,10], and numerical [11,12].

The experimental method involves too much time and a high cost. It is difficult to meet the requirements of fast iteration of multiple parameters with this method. The analytical and spring-based stiffness methods are not suitable for complex structures and cannot accurately capture the local stress around the pin hole, thus their practical value is limited. The numerical method is easily applied for studying the effects of influencing factors, such as geometric dimensions [13,14], bolt tightening force [15,16], aperture and clearance [7,17,18]. Golewski and Sadowski [19,20,21] systematically investigated the influence of the joint geometry, chamfer size, and hole diameter tolerance on hybrid single-lap and double-lap joints. Furthermore, numerical methods have some advantages, such as wide application range, low cost, good accuracy, and easy promotion in the analysis of composite bolted joints. Many studies have been carried out on the numerical method for determining pin-load distribution. Gray and McCarthy [12] established a global FE model of bolted joints to compute the pin-load distribution. The effects of bolt-hole clearance, bolt-torque, friction between laminates, and secondary and tertiary bending in laminates can also be captured well. The model was shown to be robust, accurate, and efficient. Tsai et al. [11] computed the pin-load distribution and the strength of the connecting hole. Furthermore, a nonlinear finite element analysis method is used to compute the failure strength of laminated plates. However, it is difficult to widely employ it in engineering applications due to the complexity of the modeling and the long computing time. Zhao et al. [22] proposed a modified stiffness method, considering the effect of hole tensile deformation caused by bypass load on pin-load distribution. Five kinds of multibolt joints with three to seven bolts were designed, and the pin-load ratios were predicted with the modified stiffness method. Comparisons showed that the modification can result in a more accurate prediction of pin-load distribution. Li et al. [23] established a numerical method based on the finite fracture mechanics model to predict the failure of irregular arranged multibolt composite repair. The stress distribution and stress intensity factors required in the model were obtained using a two-stage analysis strategy. In the two-stage analysis strategy, the pin-load distribution was calculated using the global analysis method. Based on the obtained pin-load, the detailed stress and stress intensity factor around individual fasteners and cutouts were determined and used to predict the failure behavior of the bolted composites. Liu et al. [24] experimentally and numerically investigated the varying pin-load distribution in double-lap and multibolt composite joints. It was found the pin-load distribution in the joint varies during the loading process and finally tends to be uniform because the damage propagating through the joint modifies the local stiffness around the holes.

When the stress distribution is obtained, the strength of the bolted joints can be evaluated by failure criteria, such as the progressive damage method [25,26,27,28], the characteristic length method [29,30,31], and the failure envelope method [32,33,34]. The characteristic length method is more widely used than the progressive damage method. Based on the test data of some specific non-notched laminates, the point stress criterion or the average stress criterion is applied to calculate the characteristic length and then extended to the strength prediction of other notched laminates. However, the value of the characteristic length is not a material property, and depends on the material type, layup, and geometry [35].

Progressive damage method is regarded as one of the best methods for strength prediction in complex composite structures. Camanho et al. [36] presented a detailed review and pointed out that the progressive damage method has advantages of determining failure mechanisms, failure propagation paths, failure modes, and both ultimate and residual strengths. Zhou et al. [25] proposed a progressive failure model based on the Puck’s criterion and cohesive zone model. The effects of geometrical parameters on the failure mode and load of T700 carbon/epoxy double-lap of bolted joints were numerically and experimentally investigated. Liu et al. [26] established three-dimensional explicit FE models combined with 3D Hashin-type initiation criteria and Camanho degradation law. The elastic loading response and overall damage profile were well predicted. Hu et al. [37] investigated the progressive failure of single-lap bolted joints of woven composites. Continuum shell elements and cohesive elements were used for the modeling of plies and interfaces, respectively. Material nonlinearities were considered by material degradation models. Choi et al. [38] predicted the failure load of a composite bolted joint with clamping force using a characteristic length method combined with the Tsai–Wu failure criteria.

Although the progressive method is widely used, it results in a long computing time. In addition, the pin-load distribution is important for the failure prediction of composite bolted joints. It has been investigated in many studies by experiments or 3D finite element models. However, few 2D models, which have a higher computational efficiency, have been studied for the pin-load distribution. In order to reduce the complexity of the procedure and save computing time, it is necessary to propose a simple analysis model, which can accurately determine the pin-load distribution ratio of fasteners and improve the efficiency of the analysis. 

**Figure 1:**
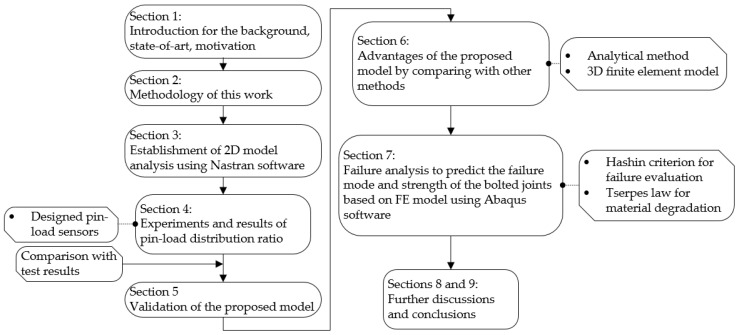
Main framework of this study.

The main object of this work is to propose a simple and efficient model with low computing cost for the determination of the pin-load distribution ratio in multibolt joints and thus provide a more convenient approach for the design of composite bolted structures. To achieve this, in this work, a two-dimensional model is presented for the pin-load analysis. Figure 1 briefly shows the main structures of this manuscript. The results obtained from the proposed model are compared with those from experiments and 3D models. Good agreement is achieved between the results from the 2D model, 3D model, and experiments, which illustrates its applicability for the pin-load distribution analysis. The analytical method fails to provide accurate results. Based on the obtained pin-load distribution from the established 2D model, a failure analysis of bolted joints is carried out.

## 2. Methodology

**Figure 2:**
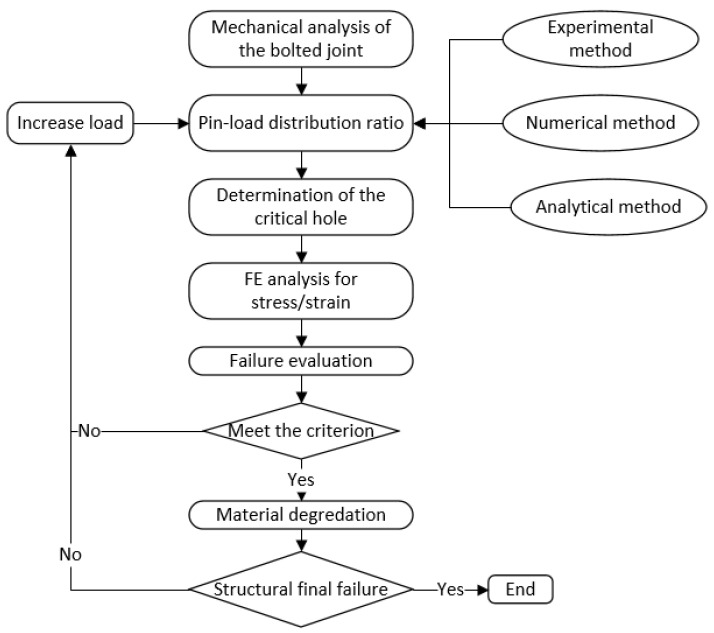
Flowchart for the failure analysis of the bolted joints.

A 2D model was established using Nastran^®^ software (version 2010, MSC Software, Los Angeles, CA, USA). In order to provide test data for the validation of the proposed model, experiments were conducted using specifically designed load sensors, which can accurately measure the values of pin-load in fasteners. The validation of the proposed model was performed by comparing the obtained values of the pin-load with the experimental values. In order to highlight the advantages of the proposed model, two other methods, the analytical method and the 3D finite element model, were investigated. Discussions of these two methods are given. Based on the determination of the pin-load distribution ratio, a failure analysis was conducted to predict the failure mode and failure strength of the bolted joints. A numerical model of the bolted joint was built using Abaqus^®^ software software (version 6.14, Dassault Systemes, Paris, France). The Hashin criterion was adopted for the failure evaluation and the degradation law proposed by Tserpes was adopted to determine the material degradation when failure occurs. Four failure modes, namely the longitudinal tension and compression failures of fibers, and the transverse tension and compression failures of matrix, were considered. The failure mechanism can be revealed by the progressive damage method and compared with the experimental results. A failure analysis of the bolted joints was performed according to the following procedures. First, we conducted a mechanical analysis of the joint and determined the external load. Secondly, according to the external load condition, we used the experimental method, the numerical method, or the analytical method to determine the pin-load distribution ratio of each fastener and identify the critical hole. Thirdly, we analyzed the detailed stress/strain field around the critical hole and used the material failure criterion to evaluate the failure. If the criterion was satisfied, we knew that failure had occurred and material degradation was required. Otherwise, we increased the load and went back to determine the pin-load distribution ratio. We repeated the above steps until the final structural failure occurred and the procedure ended. Figure 2 presents a flowchart for the failure analysis of the bolted joints.

## 3. Two-Dimensional Model for the Pin-Load Analysis

In this section, a two-dimensional finite element model is proposed for determining the pin-load distribution ratio of each fastener in multibolt joints. Descriptions of the bolted joints, the finite element model, and the calculation of the fastener’s stiffness are given.

### 3.1. Structure Description

**Figure 3:**
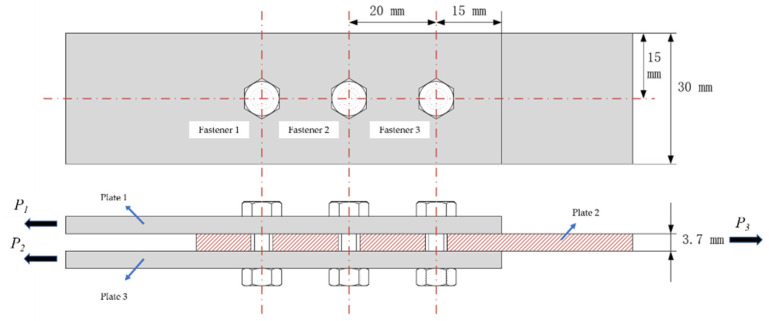
Schematic diagram and geometrical dimensions of the bolted joint.

As shown in Figure 3, the connection mode was double shearing. The composite laminated plate consisted of three connected plates with a size of 223 mm × 30 mm × 3.7 mm, the spacing of the fasteners was 20 mm, and the connected plates were made of T300 material. The material parameters of the plate are listed in Table 1, and were obtained from experiments conducted according to the corresponding test standards. The laminated plate consisted of 20 layers with each layer having a thickness of 0.185 mm, and the layer sequence was [45/0/−45/0/90/0/45/0/−45/0]_s_. The “0” direction is along the plate length direction. High-locking bolts of titanium alloy were employed for the fasteners. The elastic modulus of the Ti alloy was 110,000 MPa and the Poisson’s ratio was 0.31.

### 3.2. Finite Element Model

**Figure 4:**
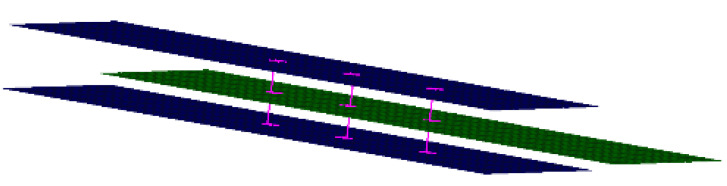
Two-dimensional finite element model of the bolted joint.

By defining the stacking sequence, the material coordinates, and the composite parameters, the CQUAD4 element was adopted to simulate the connection band. Meanwhile, the CBUSH element was used for simulating the fastener’s stiffness by defining six directional stiffnesses, i.e., axial stiffness *K*_1_, in-plane shear stiffness *K*_2_ and *K*_3_, and rotational stiffness *K*_4_, *K*_5_, and *K*_6_. The FE model built using Nastran^®^ software is presented in Figure 4. A mesh size of 2 mm was adopted in the 2D model, with 2140 elements in total. A convergence analysis was performed and showed that the mesh size is suitable for the model.

### 3.3. Calculation of the Fastener’s Stiffness

Fasteners are mainly used to transfer shear loads. When the CHUSH element is employed to simulate the fastener’s stiffness, the Huth formula is always selected according to [39], in which the in-plane shear stiffness *K*_2_ and *K*_3_ are the main factors affecting the pin-loading ratio. The formula is shown below:

(1) $$C_{i}={\left(\frac{t_{1}+t_{2}}{2d}\right)}^{a}\cdot \frac{b}{n}\left(\frac{1}{t_{1}E_{1}}+\frac{1}{nt_{2}E_{2}}+\frac{1}{2t_{1}E_{f}}+\frac{1}{2nt_{2}E_{f}}\right).$$

For the computation of the fastener’s stiffness:

(2) $$K_{i}=1/C_{i}$$

(3) $$K=\sqrt{K_{2}{}^{2}+K_{3}{}^{2}},$$

where *n* = 1, 2 for single and double shear lap, respectively. For the metal structure, *a* = 2/3 and *b* = 3.0. For the composite structure, *a* = 2/3 and *b* = 4.2. *d* is the diameter of the fastener. When the connection form is single lap, *t*_1_ and *t*_2_ are the thickness of connecting plates 1 and 2, respectively. *E*_1_, *E*_2_, and *E_f_* are the elastic modulus of connecting plates 1 and 2 and the fastener, respectively. When it is a double lap connection, *t*_2_ and *E*_2_ are the thickness and elastic modulus of the middle plate, respectively. For the structures studied here, the Huth formula is used to compute the fastener’s stiffness. In this work, *K*_2_ = 7336 N/mm, *K*_3_ = 18,740 N/mm, and in-plane shear stiffness *K* = 1.99 × 10^4^ N/mm.

## 4. Experimental Results

In order to provide test data for verifying the applicability of the above model, experimental tests were conducted on the composite bolted joints.

**Figure 5:**
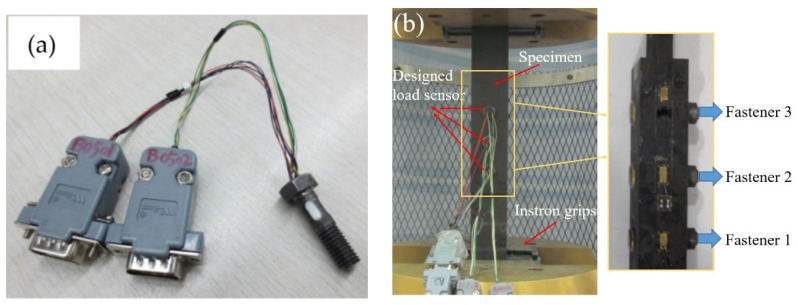
Pictures of (**a**) a specifically designed load sensor for the measurement of pin-load and (**b**) the test set-up.

**Figure 6:**
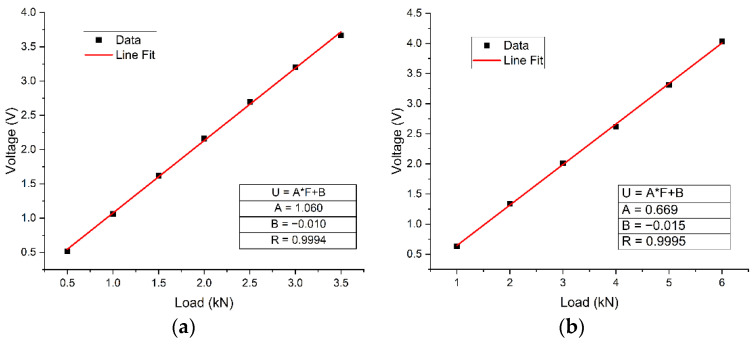
Calibration results of load sensors: (**a**) single shearing case and (**b**) double shearing case.

A double-shearing multibolt composite joint was employed, connected by three rows of fasteners. A specific load sensor, shown in Figure 5a, was designed for the measurement of the pin load in each fastener, in order to verify the proposed model. The basic material parameters are listed in Table 1. The test set-up is shown in Figure 5b. During the test, a load sensor was used to replace the bolt. A tensile load was applied at both ends of the bolted joint and the output voltage of the load sensor was measured. The pin load distribution was calculated according to the voltage–load relationship of the load sensor. Figure 6 shows the calibration results and fitting lines for the cases of single shearing and double shearing, which shows that there was a good linear relationship between the recorded voltage and the applied load.

**Figure 7:**
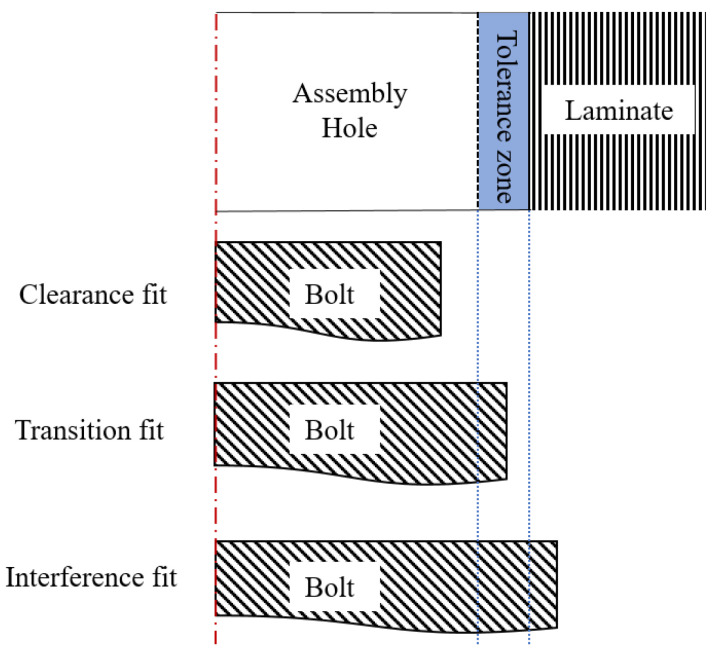
Sketch of three kinds of fit in bolted joints.

In order to measure the variation of pin-load distribution ratio of fasteners with the applied load, the pin-loads were acquired under different external loads between 3 kN and 12 kN. There are three kinds of fit for the bolted joints, as shown in Figure 7. The experimental joints in this study were not clearance fit. The diameter of the bolt was 4.76 mm, which was slightly larger than the diameter of the hole. Therefore, there was no clearance between the bolt and the hole. It should be interesting to extend related research to the joints with different degrees of clearance fit, which is plan for our future work.

**Figure 8:**
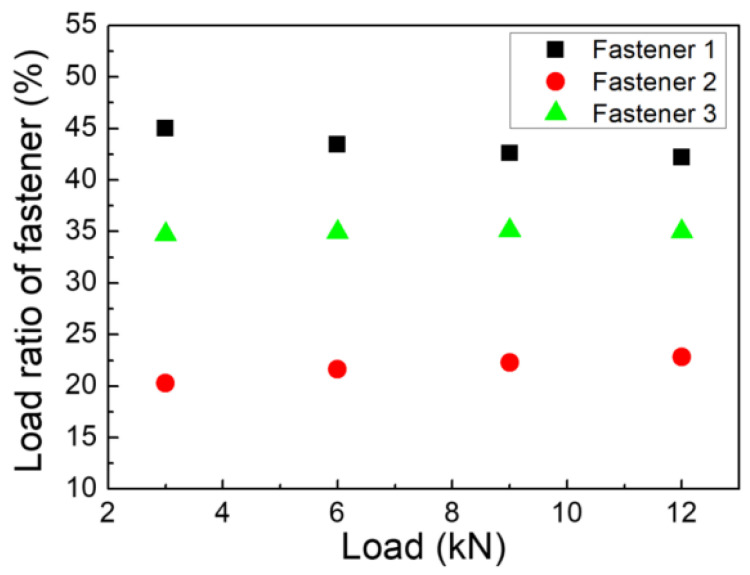
Variation of the pin-load distribution ratio of fasteners with the applied load in the three-bolt joint.

The test results for the double-shear joint with three bolts are plotted in Figure 8. It is observed that the pin-load ratio changed slightly with the increase in the external applied load, which was induced by the brittleness of the composite. This was totally different from the pin-load distribution ratio of the metal materials. Furthermore, it is known that, due to the good plasticity of the metal bolt material, the pin-load distribution ratio tends to be more evenly distributed with the increase in the applied load.

## 5. Validation of the Proposed Model

**Figure 9:**
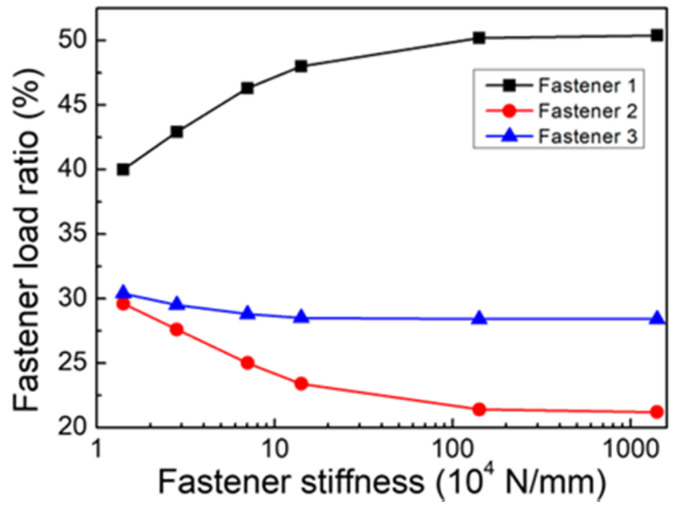
Variation of the pin-load distribution ratio of fasteners with the fastener’s stiffness.

The effects of the fastener’s stiffness on the pin-load distribution ratio were evaluated first. As shown in Figure 9, the pin-load distribution ratios of fasteners were computed under different values of the fastener’s stiffness *K*. It is clear that the larger the pin-shear stiffness, the larger the load ratio of the fastener 1. When the stiffness of the fastener reached a threshold value, the pin-load distribution ratio remained unchanged. The errors of the pin-load distribution ratio for the first row of fasteners were computed by comparing with the experimental results, which are listed in Table 2.

From Table 2, it can be seen that when the pin-shear stiffness was low, the computed pin-load distribution ratio was also relatively small. The minimum value of the pin-load ratio error was −1.0% when the pin-shear stiffness *K* was 2.83 × 10^4^ N/mm. The value of the pin-shear stiffness was 30% larger than that computed by the Huth formula. Therefore, it is reasonable to obtain the pin-load distribution ratio if the pin stiffness computed by the Huth formula is increased by 30%. In order to simplify and unify the model, the method of increasing the pin stiffness from the Huth formula was adopted for the subsequent analysis.

**Figure 10:**
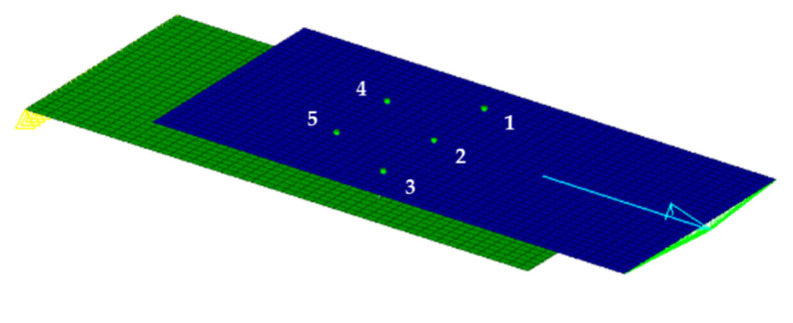
Schematic diagram of finite element model of the five-bolt joint.

**Figure 11:**
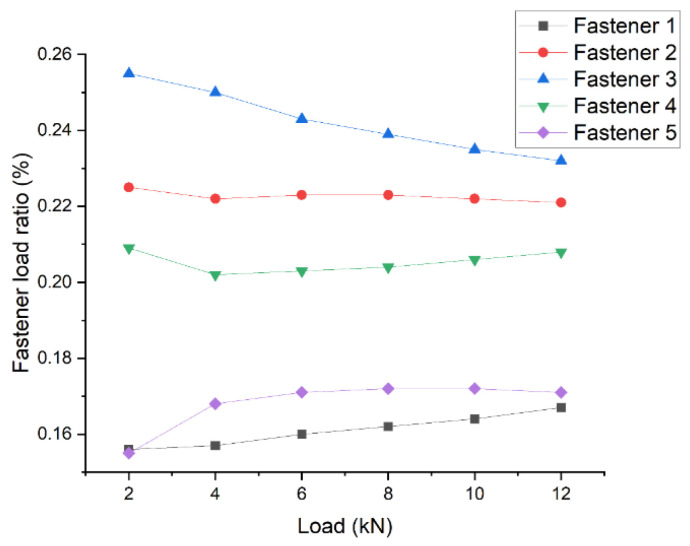
Variation of the pin-load distribution ratio of fasteners with the applied load in the five-bolt joint.

For further verification of the computational accuracy of the proposed 2D model, a single-shear model of the five-bolt joint was established, as presented in Figure 10. The load distribution ratios of fasteners 2–4 decreased with the increase in the applied load, while those of fasteners 1 and 5 exhibited the reverse trend, as shown in Figure 11. The detailed values are listed in Table 3. It can be seen that the maximum pin-load occurred on the third fastener, and the results from the proposed model were in good agreement with the test results. Therefore, it can be concluded that this proposed model can be well employed for the analysis of the pin-load distribution ratio.

## 6. Comparison with Other Methods for Determining Pin-Load Distribution

The proposed model in Section 3 can be used to determine the pin-load distribution ratio of bolted joints in a relatively simple and efficient way. In order to highlight the advantages of the proposed model, two other methods, the analytical method and the 3D finite element model, were also studied here. Their results are compared with the experimental ones.

The pin-load distribution ratio computed by the analytical method is listed in Table 4. It can be seen that the pin-load distribution ratio of the first row of fasteners was the same as that of the last row of fasteners. Furthermore, the maximum value of the pin-load distribution ratio was 34.5%, which is smaller than the 43.3% from the experimental results. This indicates that the results obtained by the analytical method were not conservative.

The main reason for this is that the bending stiffness of the laminate was ignored in the analytical method, and the laminate and the bolt were simplified to elastic elements with a certain stiffness in the loading direction. The pin-load distribution of the multirow and single-column bolted joint was computed by solving the equations of force equilibrium and deformation coordination.

**Figure 12:**
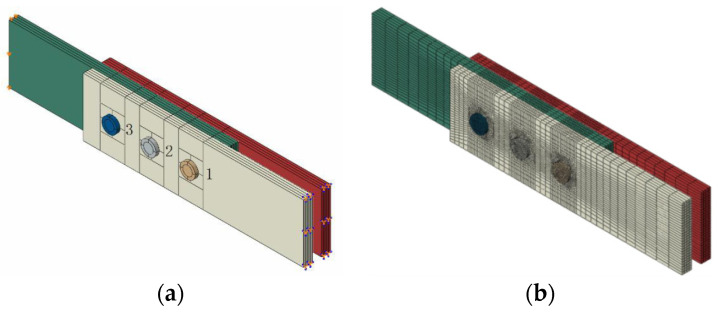
Schematic diagram of (**a**) geometrical structure and (**b**) FE model.

In a 3D finite element model, it is necessary to simulate the contacts between different structures for 3D analysis. Abaqus^®^ software was employed here. The eight-node solid element C3D8 was used to simulate the composite layer. Face-to-face contact was defined between the fastener and the plate, as well as between the plates. Furthermore, the friction coefficient between the plate and the fastener was appropriately set for the structural stress analysis. The friction coefficient was set to 0.2, which is commonly used in the engineering field. The mesh around the hole was refined in order to accurately capture the strain/stress field. A total of 50,221 elements were used in the 3D model. The Abaqus Standard solver was applied. The material adopted here was the same as in the 2D simulation. The 3D model is shown in Figure 12.

The pin-load distribution ratio is presented in Table 5. Considering there are more features reflected by the 3D model, the computed pin-load distribution ratio is more accurate than that of the 2D model. It can be seen that the loading ratio was 43.01% for the first row of fasteners, which is close to the ratio of 43.3% obtained from the experimental results. The relative error between results from the experiments and the 3D model for the pin-load distribution ratio was 0.7%, which illustrates the accuracy of the 3D model.

## 7. Failure Analysis of the Bolted Joints

Based on the determination of the pin-load distribution ratio, a failure analysis can be conducted to predict the failure mode and ultimate strength of the bolted joint. A three-row bolted joint, as described in Section 3.1, was studied here. A 3D FE analysis model was established for the capture of the realistic stress/strain field. Considering that failure usually occurs at the edge of the hole squeezed by the fastener, it is thus important to simulate this feature. Three-dimensional Hashin stress criteria [40], as in Equations (4)–(7), were adopted for the failure evaluation, and the material degradation law proposed by Tserpes et al. [41] was used for the material degradation when failure occurred.

(a) Fiber tensile failure (*σ*_1_ > 0):

(4) $${\left(\frac{\sigma_{1}}{X_{t}}\right)}^{2}+\frac{1}{S_{12}^{2}}\left(\sigma_{12}^{2}+\sigma_{13}^{2}\right)=1$$

(b) Fiber compressive failure (*σ*_1_ < 0):

(5) $$-\frac{\sigma_{1}}{X_{c}}=1$$

(c) Matrix tensile failure (*σ*_2_ + *σ*_3_ > 0):

(6) $$\frac{{\left(\sigma_{2}+\sigma_{3}\right)}^{2}}{Y_{t}^{2}}+\frac{\tau_{12}^{2}+\tau_{13}^{2}+\tau_{23}^{2}-\sigma_{2}\sigma_{3}}{S_{12}^{2}}=1$$

(d) Matrix compressive failure (*σ*_2_ + *σ*_3_ < 0):

(7) $$\begin{array}{l} \frac{1}{Y_{c}}\left[{\left(\frac{Y_{c}}{2S_{23}}\right)}^{2}-1\right]\left(\sigma_{2}+\sigma_{3}\right)+\frac{{\left(\sigma_{2}+\sigma_{3}\right)}^{2}}{4S_{23}^{2}}+\\ \frac{1}{S_{23}^{2}}(\tau_{23}^{2}-\sigma_{2}\sigma_{3})+\frac{1}{S_{12}{}^{2}}(\tau_{12}^{2}+\tau_{13}^{2})=1 \end{array}$$

where 1, 2, and 3 are the fiber direction, the transverse direction, and the normal direction, respectively. *σ*_1_, *σ*_2_, and *σ*_3_ are the stresses along the 1, 2, and 3 directions, respectively. *τ*_12_, *τ*_13_, and *τ*_23_ are shearing stresses. *X_t_* and *X_c_* are the tensile and the compressive strength along the 1 direction, respectively. *Y_t_* and *Y_c_* are the tensile and the compressive strength along the 2 direction, respectively. *Z_t_* and Z*_c_* are the tensile and the compressive strength along the 3 direction, respectively. *S*_12_, *S*_13_, and *S*_23_ are the shearing strengths. The detailed values of the parameters used in the Hashin model are listed in Table 6.

**Figure 13:**
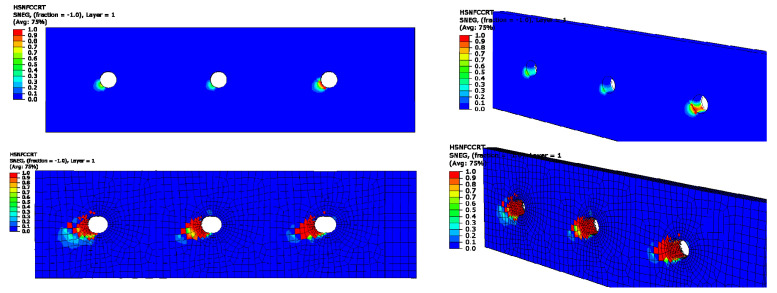
Predicted failure process of the bolted joint.

**Figure 14:**
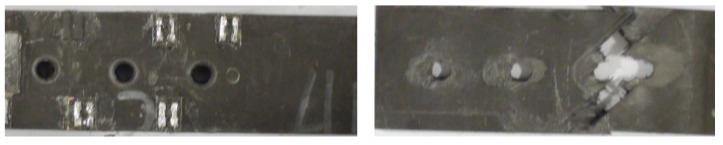
Experimentally observed failure of the bolted joint.

**Figure 15:**
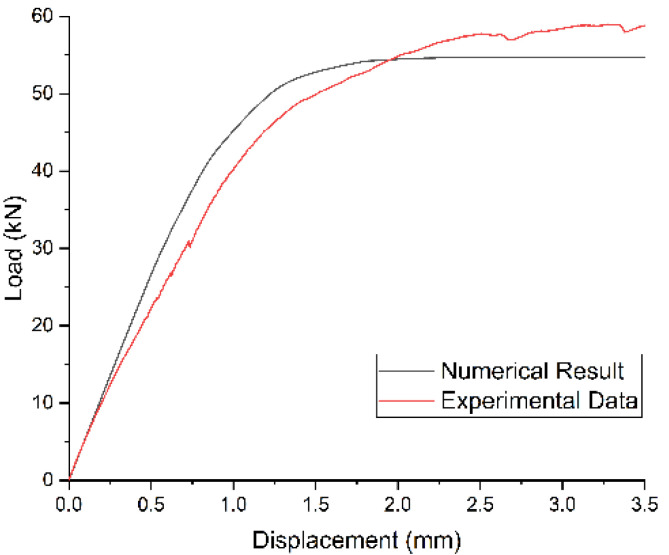
Comparison of the predicted and experimental load–displacement responses of the bolted joint.

Figure 13 presents the progressive damage process of the bolted joint. It is clear that failures of both fiber and matrix occur at the edge of the hole. The predicted damage status was consistent with the experimental results, as shown in Figure 14. The numerical load–displacement curve is plotted in Figure 15 and compared with the experimental one. Good agreement was obtained between them. The predicted failure strength was 54.7 kN, compared with the experimental value of 57.7 kN. The relative error was less than 6%, which illustrates that the established FE model can well predict the failure mode and the failure strength of bolted composite structures.

## 8. Discussion

In this work, based on the above analysis, the most dangerous position of the double shearing composite structure with three row bolts was located at the first row of fasteners, and the pin-load distribution ratio in this part was larger than for the other fasteners. Therefore, it is necessary to pay more attention to the first row of fasteners. Furthermore, the computed pin-load was relatively small and the result was not conservative when the analytical method was employed to compute the pin-load distribution of the bolted joints.

The results simulated by the 3D model were in good agreement with the actual experimental data of the composite bolted joints. In addition, the pin-load varies with the change in the fastener stiffness if the 2D model is employed to simulate the bolted joint, so it is important to accurately simulate the fastener stiffness. This study shows that the stiffness of the fasteners computed by the Huth formula was smaller than the actual test result. For ensuring the accuracy and conservatism of the computation, it was necessary to increase the stiffness of the fasteners. The computed results were in good agreement with the experimental data if the pin shear stiffness increased by 30%.

In order to predict the failure location and failure strength of the bolted structures, a 3D model using Abaqus^®^ software was employed to predict the progressive failure process, and it was easy to determine the initiation of failure. Meanwhile, the failure strength of the structure could be acquired according to the load–displacement curve, which could be used to analyze the bearing capacity of the bolted structures.

## 9. Conclusions

Bolted joints are usually the weakest parts of composite structures. Accurately predicting the joints’ strength is important for structural design and analysis. For multibolt joints, the determination of the pin-load distribution ratio is the premise of a failure analysis. In this study, a simple and efficient 2D finite element model is proposed for the determination of the pin-load distribution ratio. Experiments were conducted on three-bolt joints in order to provide test data for the model validation. In the tests, the pin-load was measured by specifically designed load sensors. The results showed that the pin stiffness has obvious effects on the calculated pin-load distribution ratios. The suitable value of the pin stiffness is higher than that calculated by the Huth formula. Based on a suitable value of pin stiffness, the results obtained from the proposed model agree well with the experimental ones, which illustrates the applicability and accuracy of the model for determining the pin-load distribution ratio. Further validation is conducted on single-shear and five-bolt joints.

Two other methods, the analytical method and the 3D FE model, were studied for comparison. There was a large error between the results computed by the analytical method and the experimental ones. The analytical method did not give conservative results. The 3D FE model can also provide good results, but it is relatively more complex and time-consuming than the proposed 2D model.

Finally, based on the obtained pin-load distribution ratio, a finite element model was established for the failure process and the failure strength prediction of bolted joints. The predicted failure modes and failure strength agreed well with the test results, which further indicates the applicability and accuracy of the proposed model. The study results are helpful for the design of a bolted joint in practical engineering.

## Figures and Tables

**Table 1:** The material parameters of the composite laminates.

Longitudinal Young’s modulus *E*_1_/MPa	131,000	Longitudinal tensile strength *X*_t_/MPa	1170
Transverse Young’s modulus *E*_2_/MPa	8110	Longitudinal compressive strength *X*_c_/MPa	859
In-plane shear modulus *G*_12_/MPa	3660	Transverse tensile strength *Y*_t_/MPa	23.6
Poisson’s ratio *ν*_12_	0.34	Transverse compressive strength *Y*_c_/MPa	139
Thickness of each layer/mm	0.15	Shear strength *S*_12_/MPa	62.2

**Table 2:** The pin-load distribution ratio for different values of the fastener stiffness.

Shear Stiffness (N/mm)	Load Ratio of Fastener 1	Test Result	Error
1.41 × 10^4^	40.0%	43.3%	−7.7%
1.99 × 10^4^	38.8%		−10.4%
2.83 × 10^4^	42.9%		−1.0%
7.07 × 10^4^	46.3%		6.8%
1.41 × 10^5^	48.0%		10.9%
1.41 × 10^6^	50.2%		15.8%
1.41 × 10^8^	50.4%		16.5%

**Table 3:** Pin-load distribution ratio in the five-bolt joint.

Fastener No.	Fastener 1	Fastener 2	Fastener 3	Fastener 4	Fastener 5
Test	16.7%	22.1%	23.2%	20.0%	17.1%
Results from 2D model	17.1%	20.9%	22.3%	19.7%	20.2%
Relative error	2.6%	−5.4%	−3.9%	−5.5%	18.1%

**Table 4:** The pin-load distribution ratios of three-row fasteners from the analytical method.

Fastener No.	Load Ratio/%
1	34.5
2	31.0
3	34.5

**Table 5:** The pin-load distribution ratio of three-row fasteners from the 3D FE model.

Fastener No.	Load Ratio/%
1	43.01
2	25.45
3	30.54

**Table 6:** Detailed values of the parameters used in the Hashin model.

Longitudinal tensile strength	Longitudinal compressive strength	Transverse tensile strength	Transverse compressive strength	Longitudinal shear strength
3259 MPa	1626 MPa	54.6 MPa	25.3 MPa	147 MPa
Transverse shear strength	Longitudinal tensile fracture energy	Longitudinal compressive fracture energy	Transverse tensile fracture energy	Transverse compressive fracture energy
102 MPa	120 J/m^2^	240 J/m^2^	0.33 J/m^2^	1 J/m^2^

## Data Availability

Data sharing is not applicable to this article.
